# Association between* CX3CR1* rs3732378 polymorphism and neovascular age-related macular degeneration in a sample of Algerian population

**DOI:** 10.22099/mbrc.2023.46767.1809

**Published:** 2023

**Authors:** Ahlem Messal, Ghania Abid, Meriem Abdi, Aicha Idder, Naima Meroufel, Faouzia Zemani-Fodil, Mostefa Fodil

**Affiliations:** 1 Laboratory of molecular and cellular biology, Université des Sciences et de la Technologie d’Oran Mohamed Boudiaf, USTO-MB, Oran, Algeria; 2 Thematic Agency for Research in Health Sciences ATRSS, Algeria; 3 Laboratory of medical genetic applied in ophthalmology, «Hammou Boutlilis» Ophthalmology Hospital, Oran, Algeria; 4 BiOSSE (Biology of Organisms: Stress, Health, Environment), Le Mans University, F-72085 Le Mans, France

**Keywords:** Neovascular age-related macular degeneration, *CX3CR1*, polymorphism, Algerian population

## Abstract

Neovascular age-related macular degeneration (nAMD) is a progressive ocular disease, responsible for central visual loss and blindness in elderly population. Increase data demonstrate that genetic factors play an important role in pathogenesis process of this disease. The aim of this study is to investigate the association between rs3732378 polymorphism in *CX3CR1* gene and nAMD in a sample of Algerian patients. This case-control study consisted of 72 patients with nAMD and 124 control subjects. DNA of participants was extracted using salting out method. Genotyping was carried out using the TaqMan real-time polymerase chain reaction method. Statistical analysis was performed by SPSS.21.0. The prevalence of the risk genotype AA was higher in the nAMD group than in control group (OR=5.02, 95% CI=1.44-17.4, P=0.011). In our sample of Algerian patients, the rs3732378 polymorphism is associated with nAMD. This result may support the role of *CX3CR1* gene in the pathogenesis of nAMD.

## INTRODUCTION

Age-related macular degeneration (AMD) is a degenerative eye disease that occurs in the central part of the retina, called the macula, and is a leading cause of central vision loss and blindness in older people. In advanced stages, AMD can develop into wet AMD, also known as exudative or neovascular (nAMD) [1]. Accumulating evidence indicates that environmental and genetic factors are involved in the pathogenesis of AMD [2]. Recently, the potential role of gene sequence variation in the development of AMD has been considered. Several studies have shown an association between AMD and various genetic polymorphisms [3, 4]. The involvement of the immune system appears to be one of the key events in the pathogenesis of AMD [5-7].

Yoichi et al., suggested that genes encoding cytokines and their receptors might play important roles in the pathogenesis of nAMD [8]. One of the most important candidate genes studied is *CX3CR1*. This gene encodes C-X3-C motif chemokine receptor 1, a transmembrane protein involved in leukocyte adhesion and migration [9] and in modulating cellular immune responses in inflammatory and viral diseases [10, 11], including coronary heart disease [12], hypertension [13], rheumatoid arthritis [14], liver disease [15], and AIDS [16].

In 2012, Fujimura et al. reported that *CX3CR1* was associated with the development of early AMD [17]. Additionally, in 2014, Falk et al. reported a significantly lower expression of *CX3CR1* on CD8+ T cells in the neovascular AMD group compared to the control group [18].

Despite these findings, the molecular mechanism explaining the involvment of the *CX3CR1* gene in the development of AMD remainsl unclear. Several single nucleotide polymorphisms (SNPs) of this gene have been reported to be associated with AMD [19, 20]. The missense variant rs3732378 located in exon 6 of this gene has been reported as an interesting candidate by many case-control studies [19-22]. These studies examined the association between the *CX3CR1* rs3732378 polymorphism andAMD risk in different ethnic groups. However, their results remain contradictory and lead to controversial conclusions [19-22]. Furthermore, most studies examining the *CX3CR1* rs3732378 polymorphism have focused on European ancestry. Since the distribution of *CX3CR1* polymorphisms and AMD risk variants differs accrossethnic groups, these *CX3CR1* gene SNPs should be tested in different populations. However, theirt effect on susceptibility to nAMD in the Algerian population has not been studied. Therefore, in the present study, we examined a previously identified *CX3CR1* (T280M) missense variant in a case-control study in a sample of the Algerian population. We wanted to demonstrate for the first time an association of the *CX3CR1* gene with the susceptibility to AMD in this population.

## MATERIALS AND METHODS

**Study population: **In our case-control study, seventy-two nAMD patients were recruited from the Department of Ophthalmology of the Sidi Bel Abes Public hospital in Algeria. In addition, we recruited one hundred twenty-four control subjects who were volunteer’s healthy people visiting the hospital for general examination. Patients and healthy controls signed an informed consent, and the study complied with the principles of the Declaration of Helsinki. Diagnosis of nAMD was confirmed by a complete ophthalmic examination including best-corrected visual acuity (BCVA) test and Snellen chart, slit-lamp examination, dilated fundus examination, fluorescein angiography (FA), and measurement of central retinal thickness (CRT) using optical coherence tomography (OCT). 

**Genotyping: **DNA was extracted from 5 ml peripheral blood of each participant using Salting Out method. The samples were genotyped using the TaqMan® real-time polymerase chain reaction method (RT-PCR). T280M genotyping assay (Assay ID: C56871) were used in an appropriate mixture containing 2X TaqMan Universal Master Mix (Thermo Fisher Scientific, France), and nuclease-free water, according to the manufacturer’s instructions. 

**Statistical analysis: **Difference of age and sex between patients and controls was calculated respectively by student’s test (t) and $x^{2}$. Hardy-Weinberg equilibrium (HWE) was also evaluated using chi square test. We examined allele and genotype distributions for the studied SNP using $x^{2}$ and logistic regression to calculate Odds Ratio (OR) and 95% confidence intervals (CI). A *P*-value less than 0.05 was considered as statistically significant. Statistical analysis were conducted by SPSS 21.0.

## RESULTS

A Total of 72 (37 males, 35 females) patients with nAMD and 124 (55 males, 69 females) control subjects participated in our study. The mean±SD age of the control and pateint groups were 62.8±8.9 and 72.0±9.0 years, respectively. 

Table 1 shows distribution of genotype and allele frequencies of *CX3CR1* rs3732378 polymorphism in patients and controls. Hardy-Weinberg equilibrium (HWE) analysis showed that genotypes were distributed homogeneously and did not deviate from HWE in the group of controls ($x^{2}$=0.32, df=1, P=0.85). Our Statistical results showed that the AA genotype was higher in patients than in controls, and it was significantly associated with nAMD risk (OR=5.02, 95% CI=1.44-17.4, P=0.011). In addition, the A allele was higher in cases compared to controls (OR=1.82, 95% CI=1.14-2.90, P=0.011) (Table1).

**Table 1:** Genotypic and allelic frequencies of the rs3732378 polymorphism among nAMD patients and controls

**Genotypes / Alleles**	**Cases (%)**	**Controls (%)**	**OR**	**95% CI**	**P**
**GG**	34 (47.2)	76 (61.3)	1.0	-	-
**GA**	29 (40.3)	44 (35.5)	1.47	0.79-2.73	0.220
**AA**	9 (12.5)	4 (3.2)	5.02	1.44-17.4	0.011
					
**G**	97 (67.4)	196 (79.0)	1.0	-	-
**A**	47 (32.6)	52 (21.0)	1.82	1.14-2.90	0.011

## DISCUSSION

AMD is a degenerative ocular disease; its prevalence increase in elder adults and is the common cause of visual impairment in developed countries [23]. In Algeria, blinding eye pathologies are a real public health problem. AMD comes in the 4 th position after cataract, glaucoma, and diabetic retinopathy with a prevalence of 2.1% (24). As a complex disease, AMD resulted from the interaction of several genetic and environmental risk factors [25- 27]. Some studies suggested that genes encoding cytokines and their receptors might play an important role in the pathogenesis of nAMD [8, 18]. One of the most studied chemokine receptor is* CX3CR1* gene, which might implicate in the pathogenesis of AMD. *CX3CR1* gene is located in chromosome 3p21.3, and contains 5 exons [9]. Several SNPs in this gene have been reported to play an important role in the exploration of the pathogenesis of nAMD in different populations. However, to our knowledge, no research has been on nAMD susceptibility in the Algerian population. Therefore, we aimed, to investigate the association of *CX3CR1* rs3732378 polymorphism with nAMD in a sample of Algerian population. 

Our results indicated a significant association of AA genotype of rs3732378 polymorphism with the risk of nAMD in our sample. This SNP was previously reported in different ethnicities; however, its impact on nAMD was controversial from population to another one. Our results in this present study are in accordance with a previous study of Yang et al., in which rs3732378 was found to be associated with an increased incidence of AMD in Chinese population [28]. In the same population, Baofeng et al., reported that A allele of rs3732378 increased the risk of AMD with the OR of 2.4 [19]. However, an inconsistent result was reported by Debra et al., who demonstrated that rs3732378 had no significant association with AMD in USA population [20]. Baofeng et al., explained the inconsistent results between Chinese and USA population by the frequencies of minor allele of rs3732378 of *CX3CR1* gene, which was much lower in Chinese (3%) than that in Caucasian population (21%) [19, 29]. However, we reported a minor allele frequency of 21%, which is comparable to Caucasian population. This difference in frequencies could be, explained by the different ethnicities, the genotyping methods and the sample size of these studies. 

In addition, two studies failed to reveal any association between the rs3732378 polymorphism and AMD in Greek and Indian populations [21, 22]. In 2015, Dan Li et al., reported in a meta-analysis that there was no significant association between the rs3732378 polymorphism and risk of AMD under all genetic models; this result was justified by the limited sample size, therefore finding on *CX3CR1* polymorphisms needs further investigation [30]. These inconsistent results could be explained by the study variation in data collecting methods, different ethnic populations, and different sample size. 

In conclusion, our study reported for the first time in a sample of Algerian population, the association of rs3732378 polymorphism in *CX3CR1* gene with increased risk of nAMD. Additional studies in different ethnic populations using standardized methodology are needed in order to confirm this association. 

## Conflict of Interest:

The authors report no conflict of interest.
